# On the Dynamics of the Adenylate Energy System: Homeorhesis vs Homeostasis

**DOI:** 10.1371/journal.pone.0108676

**Published:** 2014-10-10

**Authors:** Ildefonso M. De la Fuente, Jesús M. Cortés, Edelmira Valero, Mathieu Desroches, Serafim Rodrigues, Iker Malaina, Luis Martínez

**Affiliations:** 1 Institute of Parasitology and Biomedicine “López-Neyra”, CSIC, Granada, Spain; 2 Department of Mathematics, University of the Basque Country UPV/EHU, Leioa, Spain; 3 Unit of Biophysics (CSIC, UPV/EHU), and Department of Biochemistry and Molecular Biology University of the Basque Country, Bilbao, Spain; 4 Biocruces Health Research Institute, Hospital Universitario de Cruces, Barakaldo, Spain; 5 Ikerbasque: The Basque Foundation for Science, Bilbao, Basque Country, Spain; 6 Department of Physical Chemistry, School of Industrial Engineering, University of Castilla-La Mancha, Albacete, Spain; 7 INRIA Paris-Rocquencourt Centre, Paris, France; 8 School of Computing and Mathematics, University of Plymouth, Plymouth, United Kingdom; 9 Department of Physiology, University of the Basque Country UPV/EHU, Bilbao, Spain; University Paris South, France

## Abstract

Biochemical energy is the fundamental element that maintains both the adequate turnover of the biomolecular structures and the functional metabolic viability of unicellular organisms. The levels of ATP, ADP and AMP reflect roughly the energetic status of the cell, and a precise ratio relating them was proposed by Atkinson as the adenylate energy charge (AEC). Under growth-phase conditions, cells maintain the AEC within narrow physiological values, despite extremely large fluctuations in the adenine nucleotides concentration. Intensive experimental studies have shown that these AEC values are preserved in a wide variety of organisms, both eukaryotes and prokaryotes. Here, to understand some of the functional elements involved in the cellular energy status, we present a computational model conformed by some key essential parts of the adenylate energy system. Specifically, we have considered (I) the main synthesis process of ATP from ADP, (II) the main catalyzed phosphotransfer reaction for interconversion of ATP, ADP and AMP, (III) the enzymatic hydrolysis of ATP yielding ADP, and (IV) the enzymatic hydrolysis of ATP providing AMP. This leads to a dynamic metabolic model (with the form of a delayed differential system) in which the enzymatic rate equations and all the physiological kinetic parameters have been explicitly considered and experimentally tested *in vitro*. Our central hypothesis is that cells are characterized by changing energy dynamics (*homeorhesis*). The results show that the AEC presents stable transitions between steady states and periodic oscillations and, in agreement with experimental data these oscillations range within the narrow AEC window. Furthermore, the model shows sustained oscillations in the Gibbs free energy and in the total nucleotide pool. The present study provides a step forward towards the understanding of the fundamental principles and quantitative laws governing the adenylate energy system, which is a fundamental element for unveiling the dynamics of cellular life.

## Introduction

Living cells are essentially highly evolved dynamic reactive structures, in which the most complex known molecules are synthesized and destroyed by means of a sophisticated metabolic network characterized by hundreds to thousands of biochemical reactions, densely integrated, shaping one of the most complex dynamic systems in nature [1], [2].

Energy is the fundamental element for the viability of the cellular metabolic network. All cells demand a large amount of energy to keep the entropy low in order to ensure their self-organized enzymatic functions and to maintain their complex biomolecular structures. For instance, during growth conditions it has been observed that in microbial cells the protein synthesis accounts for 75% of the total energy, and the cost of DNA replication accounts for 2% of the energy [3], [4].

Although different nucleosides can bind to three phosphates which may serve to store biochemical energy i.e., GTP, (d)CTP, (d)TTP and (d)UTP [5], there exists a consensus that adenosine 5′-triphosphate (ATP) is the principal molecule for storing and transferring energy in cells. All organisms, from the simplest bacteria to human cells, use ATP (Mg-ATP) as their major energy source for metabolic reactions [6]–[8], and the levels of ATP, ADP and AMP reflect roughly the energetic status of the cell [7]. ATP is originated from different classes of metabolic reactions, mainly substrate-level phosphorylation, cellular respiration, photophosphorylation and fermentation, and it is used by enzymes and structural proteins in all main cytological processes, i.e., motility, cell division, biosynthetic reactions, cell cycle, allosteric regulations, and fast synaptic modulation [7]–[9].

In the living cell, practically all bioenergetic processes are coupled with each other via adenosine nucleotides, which are consumed or regenerated by the different enzymatic reactions. In fact, the most important regulatory elements involved in the coupling of catabolic and anabolic reactions are ATP, ADP and AMP [7]. The adenosine nucleotides are not only tied to the metabolic pathways involved in the cell's energetic system but also act as allosteric control of numerous regulatory enzymes allowing that changes in ATP, ADP and AMP levels can practically regulate the functional activity of the overall multienzymatic network of cell [10]–[13].

A characteristic of the temporal evolution of ATP, ADP and AMP concentrations is their complexity [14]. Extensive experimental studies have shown that metabolism exhibits extremely large and complex fluctuations in the concentrations of individual adenosine nucleotides, which are anything but stationary [14]–[16]. In fact, under normal conditions inside the cell, the time evolution of the adenosine-5′-triphosphate is subjected to marked variations presenting transitions between quasi-steady states and oscillatory behaviors [15], [16]. For instance, complex ATP rhythms were reported to occur in: myxomycetes [17], [18], neurons [19], yeast [16], embrionary cells [20], [21], myocytes [22], islet β-cells [23], [24], keratinocytes [25], hepatocytes [26], red blood cells [27] and L and MEL cells [28]. Many of these oscillations have clearly non-periodic behaviors [19], [26], and ADP and AMP also exhibit complex oscillatory patterns [29]–[31]. In addition to ATP ultradian oscillations, specific circadian rhythms have also been reported, which occur with a period close to 24 hours (the exogenous period of the Earth's rotation) [27], [32], [33].

Oscillatory behavior is a very common phenomenon in the temporal dynamics of the concentration for practically all cell metabolites. Indeed, during the last four decades, the studies of biochemical dynamical behaviors, both in prokaryotic and eukaryotic organisms, have shown that in cellular conditions spontaneous molecular oscillations emerge in most of the fundamental metabolic processes. For instance, specific biochemical oscillations were reported to occur in: free fatty acids [34], NAD(P)H concentration [35], biosynthesis of phospholipids [36], cyclic AMP concentration [37], actin polymerization [38], ERK/MAPK metabolism [39], mRNA levels [31], intracellular free amino acid pools [40], cytokinins [41], cyclins [42], transcription of cyclins [43], gene expression [44]–[47], microtubule polymerization [48], membrane receptor activities [49], membrane potential [50], [51], intracellular pH [52], respiratory metabolism [53], glycolysis [54], intracellular calcium concentration [55], metabolism of carbohydrates [56], beta-oxidation of fatty acids [57], metabolism of mRNA [58], tRNA [59], proteolysis [60], urea cycle [61], Krebs cycle [62], mitochondrial metabolic processes [63], nuclear translocation of the transcription factor [64], amino acid transports [65], peroxidase-oxidase reactions [66], protein kinase activities [67] and photosynthetic reactions [68]. In addition, experimental observations in *Saccharomyces cerevisiae* during continuous culture have shown that the majority of metabolome also shows oscillatory dynamics [69].

Persistent properties in oscillatory behaviours have also been observed in other studies, e.g., DNA sequences [70]–[71], NADPH series [72], K^+^ channel activity [73], biochemical processes [74], [75], physiological time series [76], [77], and neural electrical activity [78], [79].

Likewise, it has been observed that genomic activity shows oscillatory behavior. For instance, under nutrient-limited conditions yeast cells have at least 60% of all gene expressions oscillating with an approximate period of 300 min [80]. Other experimental observations have shown that practically the entire transcriptome exhibits low-amplitude oscillatory behavior [81] and this phenomenon has been described as a genomewide oscillation [47], [81]–[83].

At a global metabolic level, experimental studies have shown that the cellular metabolic system resembles a complex multi-oscillator system [69], [81], [83], what allows for interpretation that the cell is a complex metabolic network in which multiple autonomous oscillatory and quasi-stationary activity patterns simultaneously emerge [84]–[89].

Cells are open dynamic systems [90], [91], and when they are exposed to unbalanced conditions, such as metabolic stress, physiological processes produce drastic variations both in the concentration of the adenosine nucleotides [15], [16], [92], [93] and in their molecular turnovers [94]. Tissues such as skeletal and cardiac muscles must sustain very large-scale changes in ATP turnover rate during equally large changes in work. In many skeletal muscles, these changes can exceed 100-fold [95].

The ratio of ATP, ADP and AMP is functionally more important than the absolute concentration of ATP. Different ratios have been used as a way to test the metabolic pathways which produce and consume ATP. In 1967, Atkinson proposed a simple index to measure the energy status of the cell, defined as AEC  =  ([ATP] +0.5[ADP])/([ATP] + [ADP] + [AMP]) [96].

The AEC is a scalar index ranging between 0 and 1. When all adenine nucleotide pool is in form of AMP the energy charge (AEC) is zero, and the system is completely discharged (zero concentrations of ATP and ADP). With only ADP, the energy charge is 0.5. If all adenine nucleotide pool is in form of ATP the AEC is 1.

The first experimental testing of this equation showed that (despite of extremely large fluctuations in the adenosine nucleotide concentrations), many organisms under optimal growth conditions maintained their AEC within narrow physiological values, between AEC = 0.7 and AEC = 0.95, stabilizing in many cases at a value close to 0.9. Atkinson and coauthors concluded that for these values of AEC, the major ATP-producing reactions are in balance with the major ATP-consuming reactions; for very unfavorable conditions the AEC drops off provoking cells to die [97]–[101].

During the last four decades, extensive biochemical studies have shown that the narrow margin of the AEC values is preserved in a wide variety of organisms, both eukaryotes and prokaryotes. For instance, AEC values between 0.7 and 0.95 have been reported to occur in cyanobacteria [102], [103], mollicutes (mycoplasmas) [104], different bacteria both gram positive and gram negative as *Dinoroseobacter shibae* [105], *Streptococcus lactis* [106], *Bacillus licheniformis* [107], *Thermoactinomyces vulgaris* [108], *Escherichia coli* [109], *Myxococcus xanthus* [110] and *Myxococcus Coralloides* [111], different eukaryotic cells as zooplankton [112], algae [113], yeast [114], neurons [115], [116], erythrocytes [117], astrocytes [118], platelets [119], spermatozoa [120], embryonic kidney cells (HEK) [121], skeletal muscle [122], liver tissue [123], fungi [124], different microorganisms of mangrove soils and water (fungi, bacteria and algae) [125] and plants [126]–[128].

Studies of different species of plants, over long periods of time, have demonstrated a close relationship between AEC and cellular growth, e.g. leaf tissue collected bimonthly from *Spartina patens*, *S. cynosuroides*, *S. alterniflora* and *Distichlis spicata* showed that the adenylate energy charge peaked in spring and summer at 0.78–0.85 and then declined in late summer and early fall [129]. In the case of organisms better adapted to cold, such as winter wheat cells (*Triticum aestivum*) that are cultivated from September to December in the Northern Hemisphere, the ATP levels were shown to decrease gradually when the cells were exposed to various low temperature stresses (ice encasement at −1°C); however, even after 5 weeks of icing when cell viability was severely reduced, AEC values remained high, about 0.8 [130].

There is a long history of quantitative modelling of ATP production and turnover, dating back to Sel'kov's model on glycolytic energy production from 1968 [131], later developed by Goldbeter [132], as well as by Heinrich and Rapoport [133]. In this context, Sel'kov also published a kinetic model of cell energy metabolism with autocatalytic reaction sequences for glycolysis and glycogenolysis in which oscillations of the adenylate energy charge were observed [134].

However, the first adenylate energy system was developed by Reich and Sel'kov in 1974 [135]. This system was modeled with first-order kinetics by using ordinary differential equations.

Here, in order to further understanding of the elements that determine the cellular energy status of cells we present a computational model conformed by some key essential parts of the adenylate energy system. Specifically, the model incorporates (I) the main synthesis process of ATP for cell from ADP (ATP synthase), (II) the catalyzed phosphotransfer reaction for interconversion of adenine nucleotides (ATP, ADP and AMP) (adenylate kinase), (III) the enzymatic hydrolysis of ATP yielding ADP (kinase and ATPase reactions) and (IV) the enzymatic hydrolysis of ATP providing AMP (enzymatic processes of synthetases). The metabolic model has been analyzed by using a system of delay differential equations in which the enzymatic rate equations and all the physiological kinetic parameters have been explicitly considered and experimentally tested *in vitro* by other groups. We have used a system of delay-differential equations fundamentally to model the asynchronous metabolite supplies to the enzymes.

The numerical analysis shows that the AEC can perform transitions between oscillations and steady state patterns in a stabilized way, similar to what happens in the prevailing conditions inside the cell. The max and min values of the oscillations range within a physiological window validated by experimental data.

We finally suggest that rather than a permanent physiological stable state (*homeostasis*), the living systems seem to be characterized by changing energy dynamics (*homeorhesis*).

## Methods

Cells require a permanent generation of energy flow to keep the functionality of its complex metabolic structure which integrates a large ensemble of enzymatic processes, interconnected by a network of substrate fluxes and regulatory signals [4].

**Figure 1:**
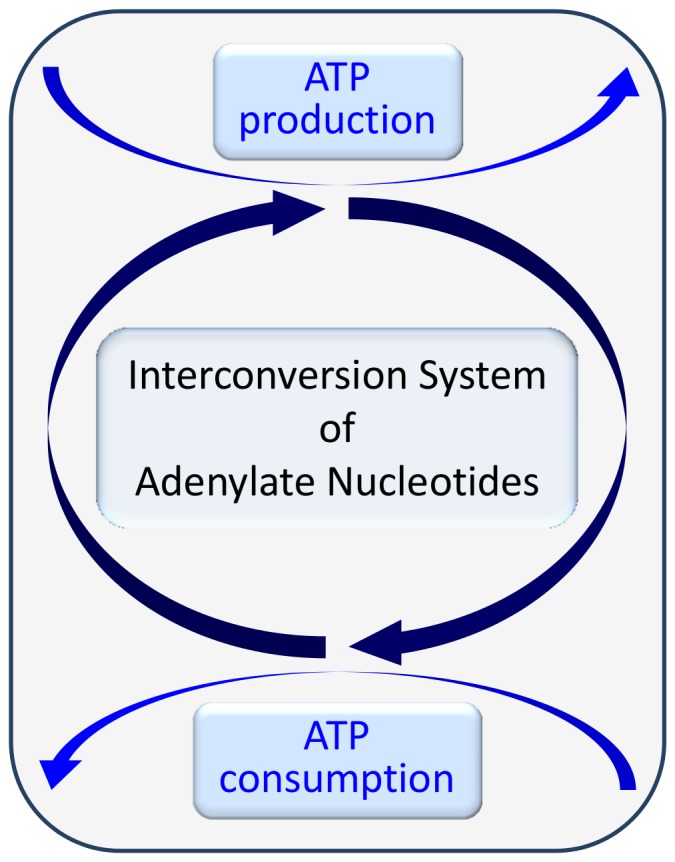
Elemental biochemical processes involved in the energy status of cells. The synthesis sources of ATP are coupled to energy-consumption processes through a network of enzymatic reactions which, interconverting ATP, ADP and AMP, shapes a permanent cycle of synthesis-degradation for the adenine nucleotides. This dynamic functional structure defines the elemental processes of the adenylate energy network, a thermodynamically open system able to accept, store, and supply energy to cells.

To understand some elements that determine the energy status of cells we have studied the dynamics of the main biochemical reactions interconverting ATP, ADP and AMP. Specifically, we have developed a model for the basic structure of the adenylate energy system which represents the fundamental biochemical reactions interconverting ATP, ADP and AMP coupled to the main fluxes of adenine nucleotides involved in catabolic and anabolic processes (Figure 1).

The essential metabolic processes incorporated into the adenylate energy model are the following:

I. First, we have assumed the oxidative phosphorylation as the main synthesis source of ATP in the cell.

As is well known, the enzymatic oxidation of nutrients generates a flow of electrons to O_2_ through protein complexes located in the mitochondrial inner membrane in eukaryotes, and in the cell intermembrane space in prokaryotes, that leads to the pumping of protons out of the matrix. The resulting uneven distribution of protons generates a pH gradient that creates a proton-motive force. This proton gradient is converted into phosphoryl transfer potential by ATP synthase which uses the energy stored in the electrochemical gradient to drive the synthesis of ATP from ADP and phosphate (P_i_) [7]. Thus, oxidative phosphorylation is the culmination of a series of complex enzymatic transformations whose final phase is carried out by ATP synthase.

Experimental studies in non-pathologic cells have shown that ATP synthase generates the vast majority of cellular energy in the form of ATP (more than 90% in human cells) [136]; consequently, it is one of the central enzymes in energy metabolism for most cellular organisms, both prokaryotes and eukaryotes. This sophisticated rotatory macromolecular machine is embedded in the inner membrane of the mitochondria, the thylakoid membrane of chloroplasts, and the plasma membrane of bacteria [137].

The overall reaction sequence for the ATP synthase is: 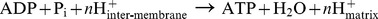
(1)where *n* indicates the H^+^/ATP ratio with values between 2 and 4 which have been reported as a function of the organelle under study [138].

II. Besides the oxidative phosphorylation, we have also considered that in optimal growth conditions a small part of ATP is generated through substrate-level phosphorylation [7].

III. Another essential metabolic process for cellular energy is the catalyzed phosphotransfer reaction performed by the enzyme adenylate kinase, which is required for interconversion of adenine nucleotides.

Almost since its discovery, about 60 years ago, adenylate kinase (phosphotransferase with a phosphate group as acceptor) has been considered to be a key enzyme in energy metabolism for all organisms [139]–[140]. This enzyme catalyzes the following reversible reaction for the interconversion of ATP, ADP and AMP: 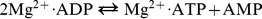
(2)


Adenylate kinase catalyzes the interconversion of the adenine nucleotides and so it is an important factor in the regulation of the adenine nucleotide ratios in different intracellular compartments, i.e. it contributes to regulate the adenylate energy charge in cells. The equilibrium will be shifted to the left or right depending on the relative concentrations of the adenine nucleotides. In contrast, ATP synthase catalyzes the *de novo* synthesis of the vast majority of ATP from ADP and Pi [136].

IV. The next catalytic process that we have considered corresponds to the enzymes implied in the hydrolysis of ATP to form ADP and orthophosphate (P_i_). The chemical energy that is stored in the high-energy phosphoanhydridic bonds in ATP is released, ADP being a product of its catalytic activity.

The basic reaction sequence for the enzymatic process is:
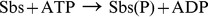
(3)where Sbs and Sbs(P) are the substrate and the product of the catalytic process, respectively. In this kind of metabolic reaction different groups of enzymes are involved, mainly kinases and ATPases. Particularly, kinases catalyze the transfer of a phosphoryl group from ATP to a different class of specific molecules, which may be also a protein. By adding phosphate groups to substrate proteins, the kinases enzymes shape the activity, localization and overall function of many proteins and pathways, which orchestrate the activity of almost all cellular processes. Up to 30% of all human proteins may be modified by a kinase activity, and they regulate the majority of cellular pathways, especially those involved in signal transduction [141]. These enzymes are fundamental for the functional regulation of the cellular metabolic network and they constitute one of the largest and most diverse gene families. The human genome contains about 500 protein kinase genes and they constitute about 2% of all human genes [141].

V. Finally, we have taken into account the ligase enzymes that catalyze the joining of smaller molecules to make larger ones, coupling the breakdown of a pyrophosphate bond in ATP to provide AMP and pyrophosphate as main products.

The basic reaction sequence for the ligases is:
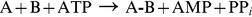
(4)


The enzymes belonging to the family of ligases involve different groups as DNA ligases, aminoacyl tRNA synthetases, ubiquitin ligase, etc. They are very important catalytic machines for anabolic processes and for the molecular architecture of the cell. Most ligases are mainly implied in the protein synthesis consuming a large part of the cellular ATP. Thus, for microbial cells, the protein synthesis accounts for 75% of the total energy during growth conditions [3], [4].

Protein synthesis uses energy mainly from ATP at several stages such as the attachment of amino acids to transfer RNA, and the movement of mRNA through ribosomes, resulting in the attachment of new amino acids to the chain. In these processes, aminoacyl tRNA synthetases constitute an essential enzyme super-family, providing fidelity of the translation process of mRNA to proteins in living cells and catalyzing the esterification of specific amino acids and their corresponding tRNAs. They are common to all classes of organisms and are of utmost importance for all cells [142]. In the present model we have considered aminoacyl tRNA synthetase as a representative enzyme of the ligases group.

**Figure 2:**
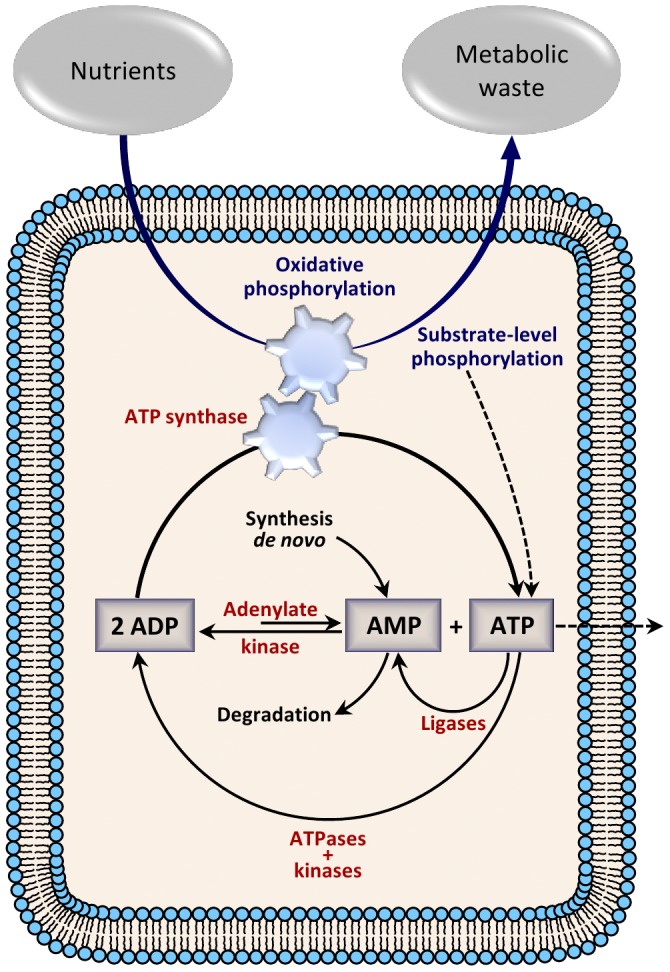
The Adenylate energy system. Oxidative phosphorylation and substrate-level phosphorylation generate ATP which is degraded by kinases (also ATPases) and ligases yielding ADP and AMP, respectively. The three adenine nucleotides are catalytically interconverted by adenylate kinase according to the needs of the metabolic system. AMP is also subjected to processes of synthesis-degradation, some AMP molecules are *de novo* biosynthesized, and a part of AMP is hydrolyzed. According to experimental observations, a very small number of ATP molecules may not remain in the adenylate reactive structure. The system (thermodynamically open) needs a permanent input of nutrients as primary energy source and a consequent output of metabolic waste. The biochemical energy system depicted in the figure represents some key essential parts of the adenylate energy system.

Figure 2 schematically shows the enzymatic processes of the ATP consuming-generating system. First, a permanent input of nutrients is considered to be the primary energy source. In the final phase of oxidative phosphorylation, the ATP synthase uses the energy stored in the proton gradient, generated by the enzymatic oxidation of nutrients, to drive the synthesis of ATP from ADP and phosphate (P_i_). The flow of protons thus behaves like a gear that turns the rotary engine of ATP synthase. Likewise, a small part of ATP is also incorporated into the system via substrate-level phosphorylation. The ATP synthesized is fundamentally consumed by two different enzymatic reactions: (i) the ligase processes which provide the system with AMP molecules and (ii) the kinase and ATPase reactions which mainly generate ADP. The interconversion of ATP, ADP and AMP is performed by the enzyme adenylate kinase, which regenerates them according to the dynamic needs of the system.

The ATP consuming-generating system is open and consequently some AMP molecules are *de novo* biosynthesized [143]; whilst a part of AMP does not continue in the reactive system due to its hydrolysis, forming adenine and ribose 5-phosphate [144]. Finally, according to experimental observations, we have considered that a very small part of ATP does not remain in the system, but is drained out from the cell [145]–[149].

We want to emphasize that the biochemical energy system depicted in Figure 2 represents some key essential parts of the adenylate energy system (see for more details the end of the “Model Section”), which constitute a thermodynamically open system able to accept, store, and supply energy to cells.

This metabolic network of crucial biochemical processes for the cell can be rewritten in a simplified way to gain a better understanding about the dynamic behavior of the model:
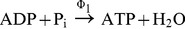
(5)

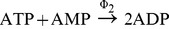
(6)

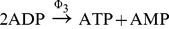
(7)

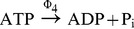
(8)

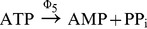
(9)

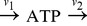
(10)

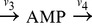
(11)where Ф_i_ (i = 1–5) are the rates of the enzymatically-catalyzed reactions (5) to (9), 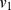 is the rate of the ATP input into the system by substrate-level phosphorylation, 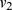 is the rate of the ATP output from the cell [145]–[149], being 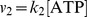, 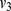 is the rate of the biosynthesis *de novo* of AMP and 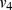 is the rate of the sink of AMP, being 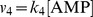. The reversible adenylate kinase reaction (2) has been described by its corresponding reactions (6) and (7) linked by a control parameter (see below for more details) allowing to move the reactive process to either of the two reactions according to the physiological needs of the system, i.e. the synthesis or the consumption of ATP or ADP. According to the stoichiometry of this set of chemical equations, there is a net consumption of ATP in the system, which can be regulated by reactions (6), (8), (9) and (10), as well as a production of AMP, which is regulated by steps (7), (9) and (11).

Although the kinetic behavior *in vivo* of most enzymes is unknown, *in vitro* studies can provide both adequate kinetic parameters and enzymatic rate functions. We have used this strategy to implement the dynamical model of the adenylate energy system. Thus, for ATP synthase we have assumed Michaelis–Menten kinetics with competitive inhibition by the product [150]. An iso-random Bi Bi mechanism has been reported for adenylate kinase kinetics [151]–[152]. We have also considered that a fraction of the adenylate kinases exhibit the balance shifted to the left and simultaneously the rest of the adenylate kinase macromolecules present a balance shifted to the right, depending their catalytic activities on the system demand. For the kinase family we have selected phosphofructokinase, whose rate equation was developed in the framework of concerted transition theory of Monod and Changeux [153], [154], and finally, for the ligase family we have chosen threonyl-tRNA synthetase, which shows Michaelis–Menten kinetics [155].

The time-evolution of the ATP consuming-generating system (Figure 2) can be described by the following three differential equations:
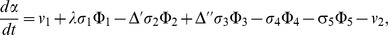


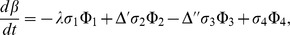


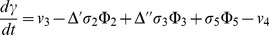
(12)where the variables α, β and γ denote the ATP, ADP and AMP concentrations respectively, 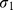,…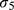 correspond to the maximum rates of the reactions (5) – (9), respectively, the nutrients are injected at a constant rate and 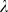 is a control parameter related to the energy level stored in the proton gradient generated by the enzymatic oxidation of input nutrients. 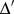 and 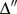 are also control parameters in the system regulating adenylate kinase activity towards the synthesis or the consumption of ADP, respectively, with 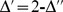
_._

The enzymatic rate functions are the following:
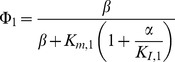
(13)

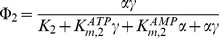
(14)

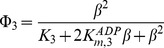
(15)

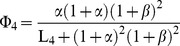
(16)

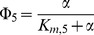
(17)where *K_m,1_*,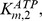 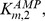 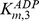
and *K_m,5_* are the Michaelis constants for each respective enzyme, *K_I,1_* is the dissociation constant of the ADP-ATP synthase complex, *K_2_* and *K_3_* are kinetic parameters of the adenylate kinase, α and β in Eq. (16) are divided by 1 µM so that this equation is dimensionally homogeneous, and L_4_ is the allosteric constant of phosphofructokinase. More details about the kinetic parameters and experimental references are given in Table 1.

**Table 1:** Values of the kinetic parameters used to simulate some of the dynamics of the adenylate energy system.

Parameter	Value	Reference
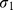	7.14 µmol s^−1^	[166]
*K_m,1_*	30 µmol	[150]
*K_I,1_*	25 µmol	[150]
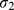	800 µmol s^−1^	[167]
*K_2_*	71000 µmol^2^	[151]
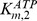	25 µmol	[151]
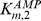	110 µmol	[151]
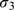	800 µmol s^−1^	[168]
*K_3_*	1360 µmol^2^	[152]
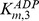	29 µmol	[152]
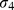	100 µmol s^−1^	[132]
*L_4_*	10^6^	[169]
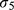	0.43 µmol s^−1^	[155]
*K_m,5_*	100 µmol	[155]

These equations are simplified expressions, but they are particularly useful in the analysis of models of dynamic behavior [154]. For simplification, we do not consider orthophosphate molecules, nor the H_2_O involved in the reaction (5), which has been omitted because the solvent has a standard state of 1M.

To study the system dynamics, the model here described has been analyzed by means of a system of delay differential equations accounting for the delays in the supplies of adenine nucleotides to the specific enzymes involved in the biochemical model.

Generally in the cellular metabolic networks the enzymatic processes are not coupled instantaneously between them. The metabolic internal medium is a complex, crowded environment [156], where the dynamic behavior of intracellular metabolites is controlled by a wide mixture of specific interactions and physical constraints mainly imposed by the viscosity of the cellular plasma, mass transport across membranes and variations in the diffusion times which are dependent on the physiological cellular context [157]–[160].

For example, there is a time-running from the instant in which ATP molecules are produced in the mitochondria until they come to the place where they are used by the target enzymes. Sometimes the spatial separations may involve long intracellular macroscopic distances. As a result of these intracellular phenomena (transport across membranes, diffusion, long macroscopic distances, interactions with the internal molecular crowded, etc.), the supply of metabolites to the enzymes (substrates and regulatory molecules) occurs in different time scales, and with different delays.

Time scales in biochemical systems mean an asynchronous temporal structure characterized by different magnitudes of metabolite supply delays associated to specific enzymatic processes.

Moreover, experimental studies have shown that metabolism exhibits complex oscillations in the concentrations of individual adenine nucleotides, with periods from seconds to several minutes [15], [16], which shape a complex temporal structure for intracellular ATP/ADP/AMP concentrations. The phase shifts in this temporal structure also originate delays in the supplies of substrates and regulatory molecules to the specific enzymes [161]–[165].

Consequently, metabolic reactions involving ATP/ADP/AMP may occur at different characteristic time scales, ranging from seconds to minutes, originating a temporal structure for intracellular ATP/ADP/AMP concentrations within the cell.

Dynamic processes with delay cannot be modeled using systems of ordinary differential equations. The different time scales can be considered with delay differential equations, which are not ordinary differential equations. In these systems, some dependent variables can be evaluated in terms of (t- r_i_) where r_i_ are the delays and t the time, and consequently the metabolite supplies to the enzymes (substrates and regulatory molecules) are not instantaneous; other dependent variables may be evaluated in terms of t (r_i_ = 0), if metabolite supplies are considered instantaneous.

According to these regards, we have analyzed our system with three delayed variables α(t-r_1_), β(t-r_2_) and γ(t-r_3_). r_1_ models the delay in the supply of ATP to its specific enzymes; r_2_ does the same for ADP and r_3_ for AMP. Nevertheless, we have assumed that ATP concentration (α(t)) in the equation corresponding to ATP synthase (Eq (18)) is not delayed, as this product formation can be considered instantaneous with respect to the competitive inhibition of the enzyme by the same ATP. Likewise, since the adenylate kinase enzyme is reversible, the ADP formed from ATP and AMP in the reaction (6) is used by the reaction (7) in the same place, and therefore, we have also considered that ADP concentration (β (t)) is not delayed in this process (Eq (20)).

Therefore, the adenylate energy system exhibits several time scales and we have used the system of delay-differential equations to model the asynchronous metabolite supplies to the enzymes. In some processes it can be considered that the substrate or regulatory molecules instantly reach the enzyme and in other processes there are delays for substrate supplies to them.

According to these kinds of dependent variables in the system, the enzymatic rate functions are written as follows:
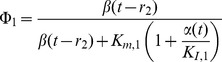
(18)

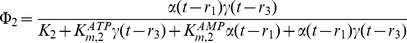
(19)

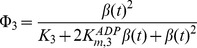
(20)

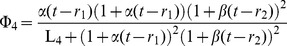
(21)

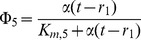
(22)


Our differential equations system with delay (12) takes the following particular form, up to a permutation of the indexes of the variables:
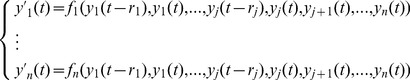
(23)where the dependent variable is a n-dimensional vector of the form 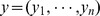, *t* being the independent variable. In system (23), the derivatives of 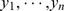, evaluated in *t*, are related to the variables 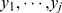, where each 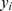 with 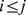 appears evaluated in 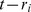, being 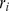 the corresponding delay, and might appear evaluated also in t, and the derivatives are also related to the variables 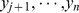 evaluated in t.

Unlike ODE systems, in delayed differential equations, in order to determine a particular solution, it is necessary to give the initial solution in the interval 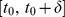 with 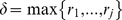. That involves the consideration, in the solution of the system, of the function 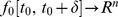 called initial function. It can be observed therefore that infinite degrees of freedom exist in the determination of the particular solutions.

Since in our system simple oscillatory behavior of period 1 emerges from numerical integration, an acceptable approximation to the initial function is a periodic solution.

In the system described by (23), it is possible to take the initial function 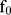 equal to any 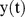 and, in particular, it can be a periodic function.

With this type of systems, it is possible to take into account dynamic behaviours related to parametric variations linked to the independent variable. The parametric variations 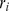 affect the independent variable; they represent time delays and can be related to the domains of the initial functions.

Table 1 shows the values of the kinetic parameters involved in the system chosen to run the model. All of these values have been obtained from *in vitro* experiments reported in the scientific literature and they are within the range of the values published in the enzyme database Brenda (http://www.brenda-enzymes.info/). For these values, the preliminary integral solutions of the differential equations system (12) show a simple oscillatory behavior of period 1 and as an approximation we have assumed that the initial functions present simple harmonic oscillations in the following form:
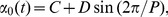
(24)

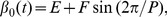
(25)

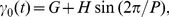
(26)with C = 6 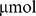, D = 2 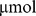, E = 4 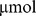, F = 1 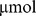, G = 7 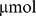, H = 3 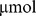 and P = 200 s. The other parameter values used were 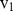 = 35
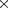
10^−3^ 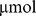 s^−1^, 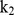 = 9
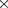
10^−5^ s^−1^, 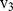 = 1.4 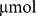 s^−1^, 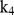 = 0.69 s^−1^, 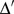 = 1.98, r_1_ = 5 s, r_2_ = 27 s and r_3_ = 50 s.

In this paper, we have studied the dynamic behavior of the system under two parametric scenarios:

- In Scenario I, 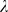 is the control parameter, which is related to the energy level stored in the proton gradient generated by the enzymatic oxidation of input nutrients. This scenario represents the main analysis of the paper, and the values used for the kinetic parameters involved in the model are those set out above.

- In Scenario II, the delay r_2_ is the control parameter, modelfing the time constants for the time delays of ADP, with 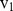 = 3
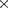
10^−3^ 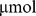 s^−1^, 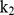 = 2
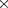
10^−4^ s^−1^, 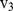 = 2.1 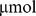 s^−1^, 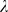 = 1.09, r_1_ = 3 s, and all other parameters as indicated in Scenario I.

The extracellular ATP concentration [145]–[149] is considerably much lower than its intracellular concentration [147], which makes accurate quantification of extracellular levels of ATP an extremely difficult task. Therefore, 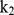 (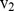 = 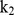 [ATP]) must be a sufficiently small value. The values of 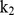 here used have been: 9×10^−5^ s^−1^ in Scenario I and 2×10^−4^ s^−1^ in Scenario II. If we now take an intermediate value for the ATP concentration, 10 nmol for example, the following data are obtained: 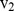 = 9×10^−7^ µmol s^−1^ under Scenario I, and 2×10^−6^ µmol s^−1^ under Scenario II, which are significantly lower than the values considered for 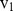 (the rate of the ATP input into the system by substrate-level phosphorylation): 3.5×10^−2^ µmol s^−1^ under Scenario I, and 3.0×10^−3^ µmol s^−1^ under Scenario II.

In this paper, we have studied the bifurcation analysis for the two control parameters (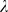 and r_2_) here considered (Scenarios I and II, resp.). Further future studies, beyond the scope of the present work, might consider including other control parameters to understand how the stability of the solutions change along parameters space. Furthermore, the presence of “molecular noise” might also be included as a possibility to achieve non-periodic variability in the ATP/ADP/AMP oscillations.

An important feature of metabolism is the wide range of time scales in which cellular processes occur.

Generally enzymatic reactions take place at high speed e. g., carbonic anhydrase has a turnover number (k_cat_) of 400,000 to 600,000 s^−1^ [170] and the turnover number for RNA polymerase II is less rapid, about 0.16 s^−1^ [171].

However, many cellular processes occur on a time scale of minutes. For instance, studies in glucose-limited cultures by up- and downshifts of the dilution rate in *Escherichia coli* K-12 have shown time delays of minutes in the metabolic mechanisms involved in the dynamics of the adenylate energy charge exhibiting drastic changes within 2 min after the nutrients dilution [109]. Intracellular concentrations of the adenine nucleotides and inorganic phosphate may present sustained oscillations in the concentrations of the adenine nucleotides with periods around a minute which can originate large temporal variations in the supplies of these substrates and regulatory molecules to the specific enzymes [30]. In addition to the temporal oscillations, sustained chemical redox waves (NAD(P)H− NAD(P)+) are a rather general feature of some cells [90] which may exhibit qualitative changes with wavefronts traveling in opposite directions within ≈2 min after the start [172].

It is also known that ATP can evoke fast currents by activation of different purinergic receptors expressed in the plasma membranes of many cells [173]. However, ATP exposure for several minutes can lead to the formation of a high conductance pore permeable for ions and molecules up to 900 Da [174], [175]. The activation of some kinases, such as MAPK, occurs with a time scale of minutes [176], [177]. Furthermore fructose-2,6-bisphosphate levels are also regulated through cyclic-AMP-based signalling, which occurs on the timescale of minutes [178].

According to these experimental observations, we have analyzed the dynamic behavior of the adenylate system taking into account both instantaneous substrate input conditions and delay times for metabolite supplies, between 1 to 120 seconds, which covers a wide range of cellular physiological processes.

The numerical integration of the system was performed with the package ODE Workbench, developed by Dr. Aguirregabiria which is part of the Physics Academic Software. Internally this package uses a Dormand-Prince method of order 5 to integrate differential equations (http://archives.math.utk.edu/software/msdos/diff.equations/ode_workbench/.html).

The use of differential equations in the study of metabolic processes is widespread nowadays and different biochemical regulation processes have been quantitatively analyzed using time delayed simulations, e.g., in the phosphorylation–dephosphorylation pathways [179], in the endocrine metabolism [180], in the Lactose Operon [181], in the regulation of metabolic pathways [182], in cell signaling pathways [183], and in metabolic networks [184].

Finally, we want to again emphasize that our model only represents some key essential parts of the adenylate energy system. As has previously been indicated, each living cell is essentially a sophisticated metabolic network characterized by hundreds to thousands of biochemical reactions, densely integrated, shaping one of the most complex dynamic systems in nature. The cellular metabolic network functionally integrates all their catalytic processes as a whole. For instance, in a cellular eukaryotic organism the systemic metabolic network includes the enzymatic reactions linked to the plasma membrane, the catabolic and anabolic processes of cytoplasm, the metabolism developed by organelles and subcellular structures, the processes of cell signaling, the adenylate energy system, the metabolism of the nuclear membrane and the nucleoplasm, the enzymatic processes for genetic expression, etc.

A fundamental property of this cellular metabolic network is their modularity. Metabolism is organized in a modular fashion and the emergence of modules is a genuine characteristic of the functional metabolic organization in all cells [185], [186].

Energy is the essential element for the viability of the cellular metabolic network, and practically all bioenergetic processes are coupled with each other via adenosine nucleotides, which are consumed or regenerated by the different enzymatic reactions of the network.

The adenosine nucleotides also act as allosteric control of numerous regulatory enzymes allowing that changes in ATP, ADP and AMP levels can practically regulate the functional activity of the overall metabolic network of cell [10]–[13].

Accordingly, the cellular energetic system is an integral part of the systemic metabolic network and also shapes a super-complex dynamical system which consists of thousands of biochemical reactions.

In addition, the cellular energy system is involved as well in the set of catabolic and anabolic reactions of the systemic metabolism exhibiting specific processes, e.g., the oxidative phosphorylation, the glycolytic metabolism and other catalytic reactions of substrate-level phosphorylation, the regulatory modular sub-networks of adenosine nucleotide signals, the AMPK system which acts as a metabolic master switch, the degradation processes of the adenosine nucleotides, the allosteric and covalent modulations of enzymes involved in bioenergetic processes, the role of AMP, AMPK and adenylate kinase in nucleotide-based metabolic signaling, the principles of dissipative self-organization of the bioenergetic processes and the significance of metabolic oscillations in the adenosine nucleotide propagation inside the cell.

## Results

To understand the dynamics of the main enzymatic reactions interconverting the adenine nucleotides we have analyzed a biochemical model for the adenylate energy system using the system of delay differential equations (12) to account for the asynchronous conditions inside the cell.

### Scenario I

Scenario I represents the fundamental analysis of the paper, being 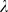 the main control parameter, which models the energy level stored in the proton gradient generated by the enzymatic oxidation of input nutrients, and therefore, represents the modifying factor for the ATP synthesis in the system due to substrate intake.

**Figure 3:**
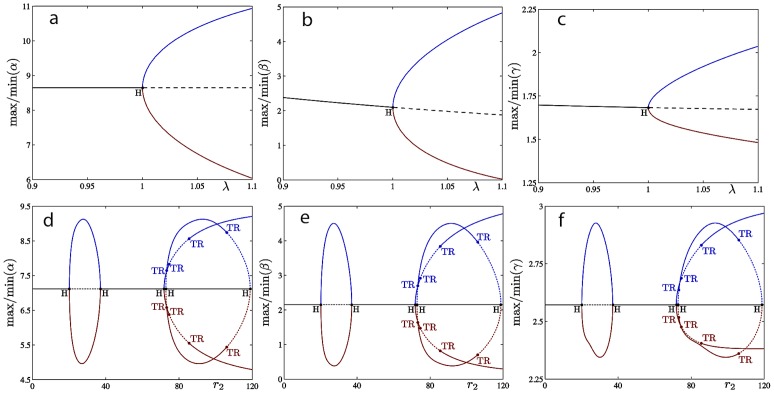
Numerical analysis for the model of the adenylate energy system. a–c: (cf. Scenario I in text) In y-axis we are plotting the max and the min of the different variables α, β and γ. For situations with no oscillations (stable fixed point colored in solid black lines) the max and the min are coincident. For situations with oscillations, the max and the min of the oscillations are plotted separately; in blue we are coloring the max of the oscillation, in red, its minimum value. 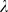 is the control parameter. The numerical integration shows simple solutions. For small 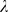 values (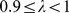) the adenine nucleotide concentrations present different stable steady states which lose stability at a Hopf bifurcation at 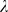
∼1. For 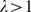, the attractor is a stable limit cycle. d–f: (Scenario II) The delay r_2_ is the control parameter. The numerical bifurcation analysis reveals that the temporal structure is complex, emerging 5 Hopf bifurcations as well as a secondary bifurcation of Neimark-Sacker type. Two pairs of Hopf bifurcations are connected in the parameter space. A third supercritical Hopf bifurcation occurs at r_2_∼71.94, rapidly followed by another Hopf bifurcation, subcritical, at r_2_∼72.83. This marks the beginning of the region where the system is multi-stable. The last Hopf bifurcation, born at r_2_∼72.83, which is subcritical exhibiting the presence of several Torus bifurcations, occurs on a branch of limit cycles when a pair of complex-conjugated Floquet multipliers, leave the unit circle. Branches of stable (resp. unstable) steady states are represented by solid (resp. dashed) black lines; branches of stable (resp. unstable) limit cycles are represented by the max of the oscillation in blue and the minimum in red and by solid (resp. dashed). Hopf bifurcation points are black dots labeled H; Torus bifurcation points are blue dots labeled TR. The bifurcation parameters 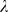 (Scenario I) and r_2_ (Scenario II) are represented on the horizontal axis. The max and min values of each variable are represented on the vertical axis.

The numerical integration illustrated in Figure 3a-c shows that the temporal structure of the biochemical model is simpler than Scenario II (see below). At small 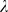 values, for 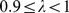
the adenine nucleotide concentrations display a family of stable steady states (notice that 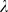 = 0.9 represents a 10% reduction of the ATP synthesis). These steady states lose stability at a Hopf bifurcation detected numerically for 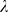
∼1 which corresponds to a normal activity of ATP synthase with a maximum rate of 7.14 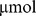 s^−1^ [166]. For values of 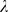 bigger than 1 the attractor of the system is a stable limit cycle (therefore, the Hopf bifurcation is supercritical). Concretely, the amplitude of adenine nucleotide oscillations augments as 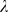 increases, e. g., for 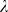 = 1.02, which represents a 2% of increment in ATP synthesis, the adenine nucleotides exhibit new oscillations with amplitude values of 2.36 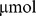 (ATP), 2.21 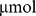 (ADP) and 0.24 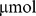 (AMP). With an 8% of increase in the ATP synthesis (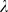 = 1.08) the amplitudes show higher values, namely 4.47 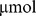 (ATP), 4.41 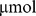 (ADP) and 0.5 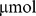 (AMP). Finally, when activity reaches a 10% increase (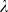 = 1.1) the three dependent variables of the metabolic system oscillate with higher amplitude concentrations: 4.92 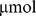, 4.84 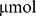 and 0.55 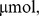 respectively.

**Figure 4:**
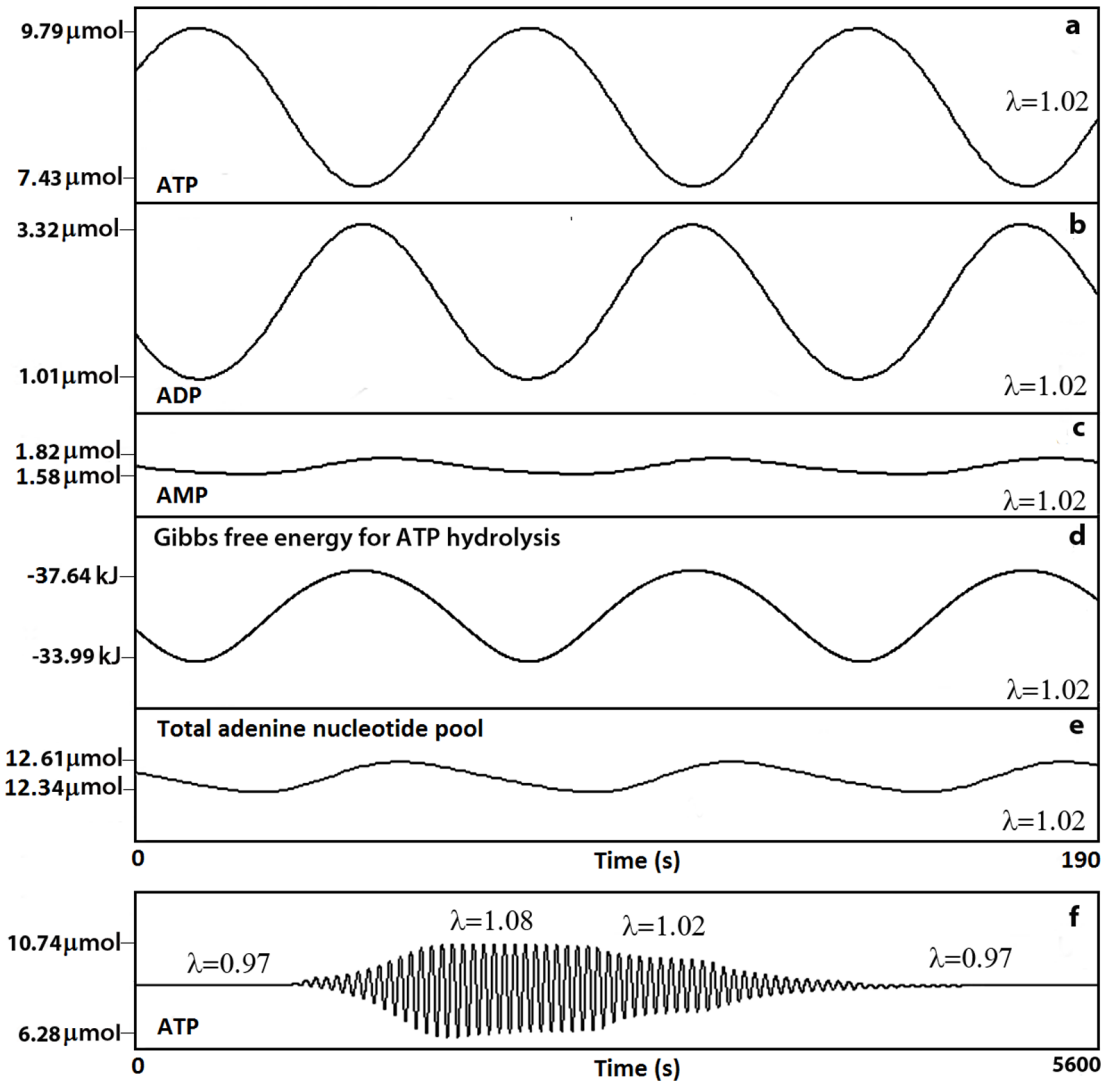
Dynamical solutions of Scenario I. For 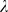 = 1.02 (normal activity for the ATP synthesis), periodic oscillations emerge. (a) ATP concentrations. (b) ADP concentrations. (c) AMP concentrations. (d) The Gibbs free energy change for ATP hydrolysis to ADP. (e) The total adenine nucleotide (TAN) pool. It can be observed that ATP and ADP oscillate in anti-phase (the ATP maximum concentration corresponds to the ADP minimum concentration). Likewise, it is noted that the total adenine nucleotide pool shows very small amplitude of only 0.27 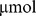 and a period around 65 s. (f) ATP transitions between different periodic oscillations and a steady state pattern for several values of 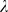
(0.97, 1.08, 1.02, 0.97). Maxima and minima values per oscillation are shown in y-axis.

Figure 4 shows three time series belonging to ATP, ADP and AMP (panels a, b and c, respectively), for 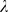 = 1.02. The largest oscillation values correspond to ATP (max = 9.79 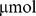 and min = 7.43 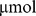) followed by ADP (max = 3.32 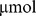 and min = 1.01 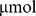) and finally, AMP which oscillates with a low relative amplitude (max = 1.82 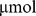 and min = 1.58 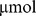). We have also observed that ATP oscillates in anti-phase with ADP and consequently the maximum concentration of ATP corresponds to the minimum concentration of ADP.

In most metabolic processes, ATP (Mg-ATP) is the main energy source for biochemical reactions and its hydrolysis to ADP or AMP releases a large amount of energy. To this respect, we have estimated the Gibbs free energy change for ATP hydrolysis (to ADP) under an emergent oscillatory condition of the system, applying the known equation 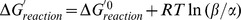. The change of the standard Gibbs free energy for this reaction was previously evaluated by Alberty and co-workers [187] obtaining a value of −32 kJmol^−1^ under standard conditions of 298 K, 1 bar pressure, pH 7, 0.25 M ionic strength and the presence of 1 mM Mg^2+^ ions forming the ATP.Mg^2+^ complex, which has different thermodynamic properties than free ATP and, it is closer to physiological conditions.

Under these conditions, Figure 4d shows the values of Gibbs free energy change of ATP hydrolysis for 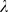 =  1.02 which corresponds to a normal activity for ATP synthesis. The resulting values for the oscillatory pattern were more negative than the standard value with a maximum and a minimum of −37.64 kJmol^−1^and −33.99 kJmol^−1^, meaning that the hydrolysis of ATP releases a large amount of free energy that can be captured and spontaneously used to drive other energetically unfavorable reactions in metabolism.

The total of adenine nucleotides is another relevant element in the study of cellular metabolic processes. Different experimental observations have shown that changes in the size levels of the adenine nucleotide pool occur under different physiological conditions [188]. We have estimated the total adenine nucleotide (TAN) pool as [ATP] + [ADP] + [AMP], and Figure 4e shows for 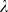 = 1.02 an emergent oscillatory behavior for TAN with a maximum of 12.61 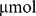 and a minimum of 12.34 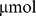, i.e., a little amplitude of only 0.27 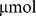 and a period of 65 sec.

Likewise, we have observed that the sum of ATP and ADP concentrations exhibits very small range. So, for 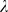 = 1.02, the amplitude is 93 nmol and for 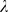 = 1.1 it is 202 nmol (data not shown in the Figure).

Figure 4f illustrates ATP transitions between different periodic oscillations and a steady state pattern for several values of 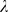
(0.97, 1.08, 1.02, 0.97).

**Figure 5:**
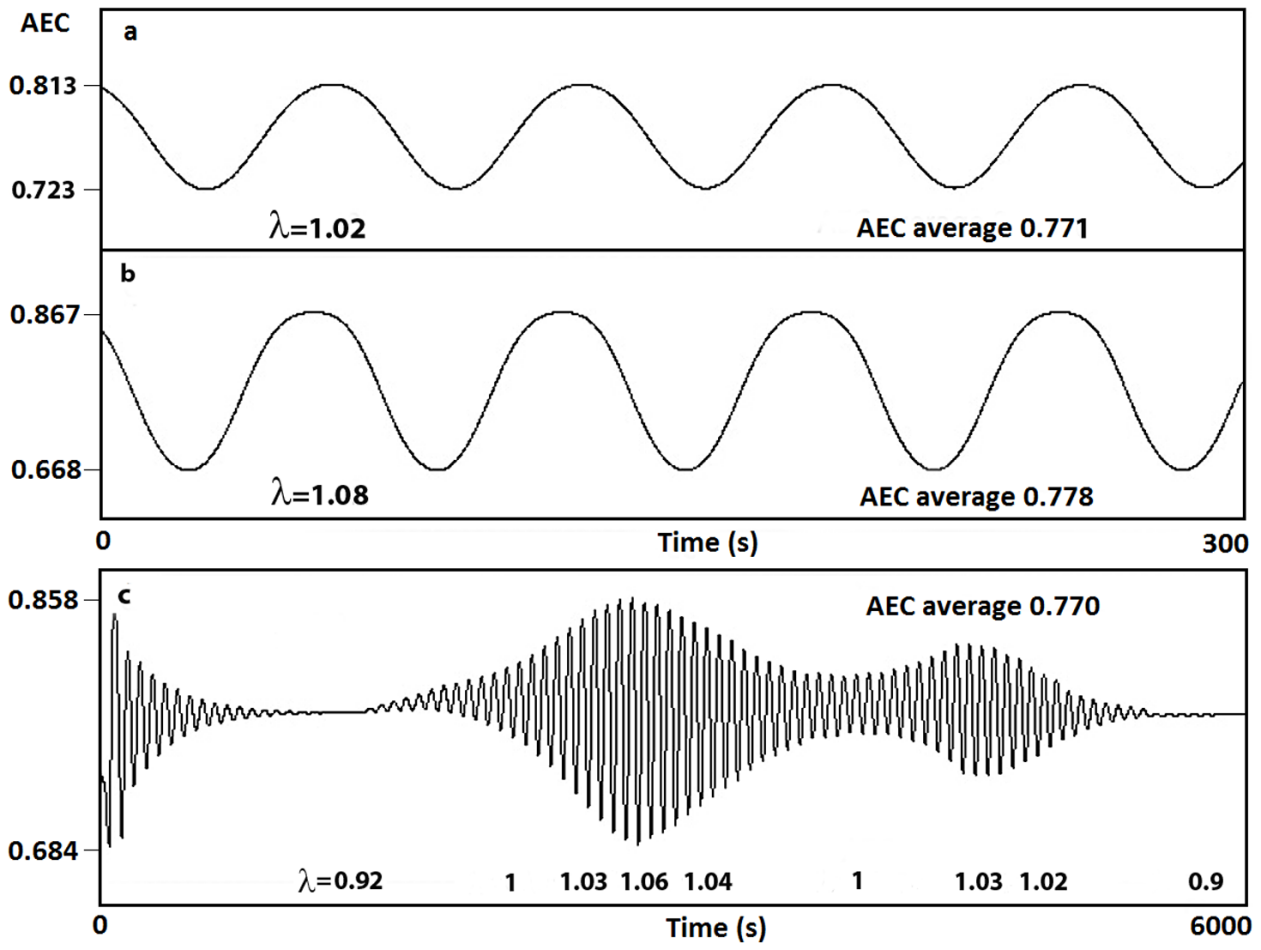
Emergence of oscillations in the AEC (Scenario I). Different oscillatory behavior appears when varying 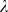, the modifying factor for the ATP synthesis. (a) For 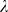 = 1.02 (normal activity of ATP synthesis) the AEC periodically oscillates with a very low relative amplitude of 0.090. (b) At higher values of ATP synthesis (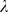 = 1.08) large oscillations emerge with a amplitude of 0.199. (c) AEC transitions between different periodic oscillations and steady state patterns for several values of 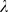 (0.92, 1, 1.03, 1.06, 1.04, 1, 1.03, 1.02, 0.9).

Next, to analyze the dynamics of the energetic status of the system we have calculated the energy charge level. Figure 5 shows different oscillatory patterns for AEC. For 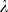 = 1.02 the AEC periodically oscillates with a low relative amplitude of 0.09 (max = 0.813 and min = 0.723) (Figure 5a). At higher values of ATP synthesis (an increment of 8%) larger oscillations emerge (max = 0.867 and min = 0.668) (Figure 5b).

Finally, Figure 5c illustrates AEC transitions between different periodic oscillations and steady state patterns for several arbitrary values of 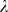 (0.92, 1, 1.03, 1.06, 1.04, 1, 1.03, 1.02, 0.9) and arbitrary integration times. All the oscillatory patterns for the energy charge maintain the AEC average within narrow physiological values between 0.7 and 0.9.

**Figure 6:**
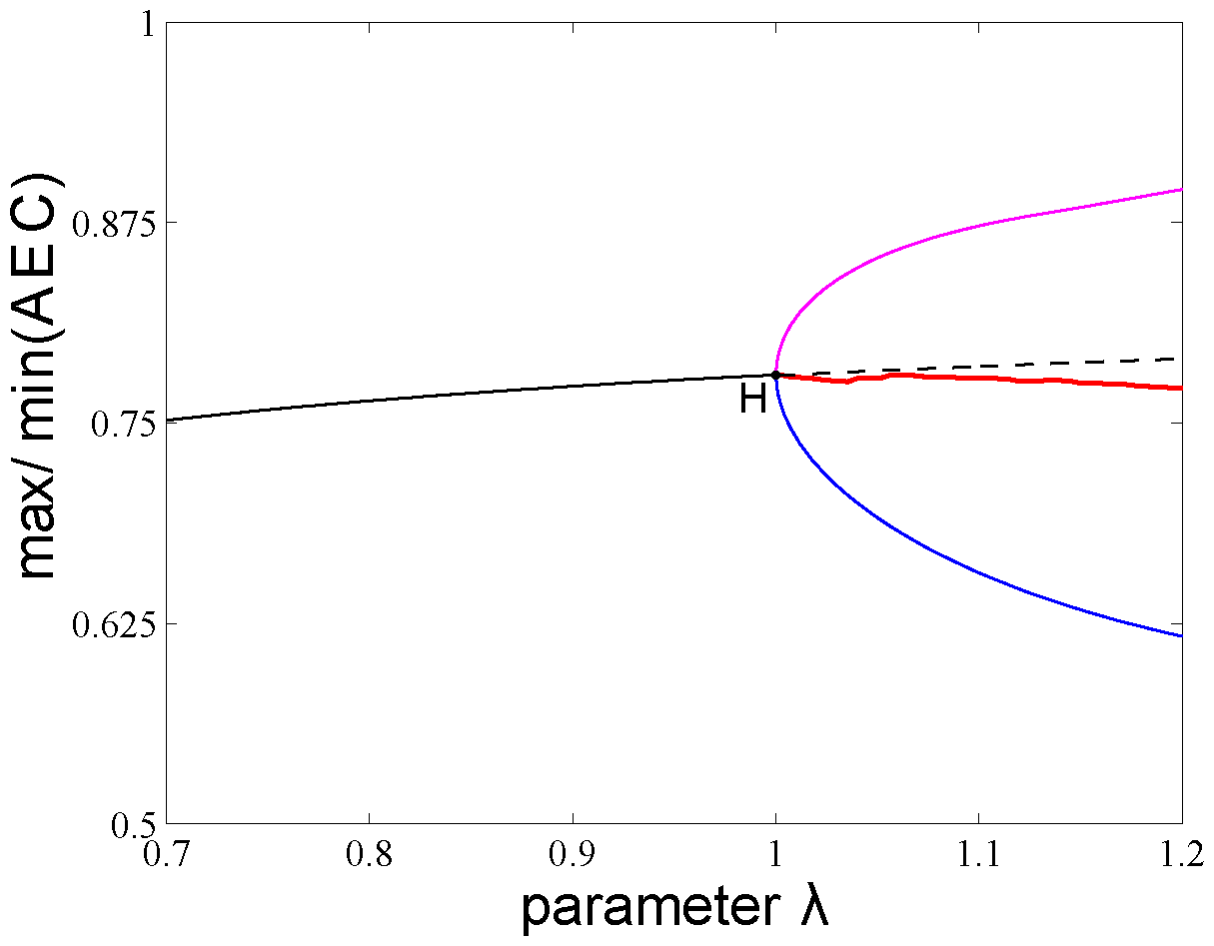
Robustness analysis for the adenylate energy charge (AEC) across different modeling conditions. In y-axis we have plotted the max and the min of the AEC. For situations with no oscillations stable fixed are point colored in solid black lines. In x-axis we have plotted the 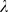 control parameter, which models the energy level stored in the proton gradient generated by the enzymatic oxidation of input nutrients. From left to right, we can see that the system has a fixed point solution which is stable for 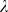
<1 (black solid line) and becomes unstable for 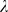
>1 (black dashed line), i.e., there is a Hopf bifurcation (H) at 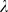
∼1. For 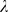
>1, the limit cycle solution becomes stable, in magenta (blue) we have colored the max (min) of the oscillations. In red, we are coloring the average AEC value of the oscillations. For 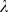
<1, the AEC values range from 0.752 and 0.779, and for 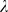
>1, the AEC average value between the maximum and minimum per period range from 0.768 to 0.756. At very small 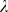 values, for 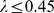 the AEC exhibits values below 0.6 (Figure 7). The AEC does not substantially change during the simulations indicating that it is strongly buffered against the changes of the main control parameter of the system.

Figure 6 shows a robustness analysis of the system in which the values of the adenylate energy charge (AEC) do not substantially change when 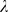, the main control parameter, is heavily modified (a 50% of its value) indicating that AEC is strongly buffered.

Thus, at small 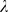 values, for 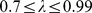, the AEC displays a family of stable steady states and the AEC values range from 0.752 to 0.779 (notice that 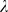 = 0.7 represents a 30% reduction of the ATP synthesis). These steady states lose stability at a Hopf bifurcation for 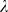
∼1 and the AEC exhibits oscillatory behaviors of period 1, being the average between the maximum and minimum of 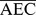 = 0.769. Notice that 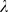 = 1 corresponds to an optimal activity of ATP synthase with a maximum rate of 7.14 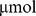
s^−1^ [166].

As expected, the maximum and minimum per period get bigger as 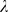 increases, and for 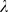 = 1.2 the AEC maximum per oscillation reaches 0.896 and the 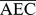
decreases to 0.769 (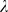 = 1.2 represents a 20% increase in activity of the optimal ATP synthesis).

This robustness analysis of the system for a perturbation of 50% in the 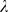 values show that in the stable steady states the AEC values range from 0.752 to 0.779 and in the stable periodic behaviors the AEC average between the maximum and minimum per period ranges from 0.768 to 0.756.

**Figure 7:**
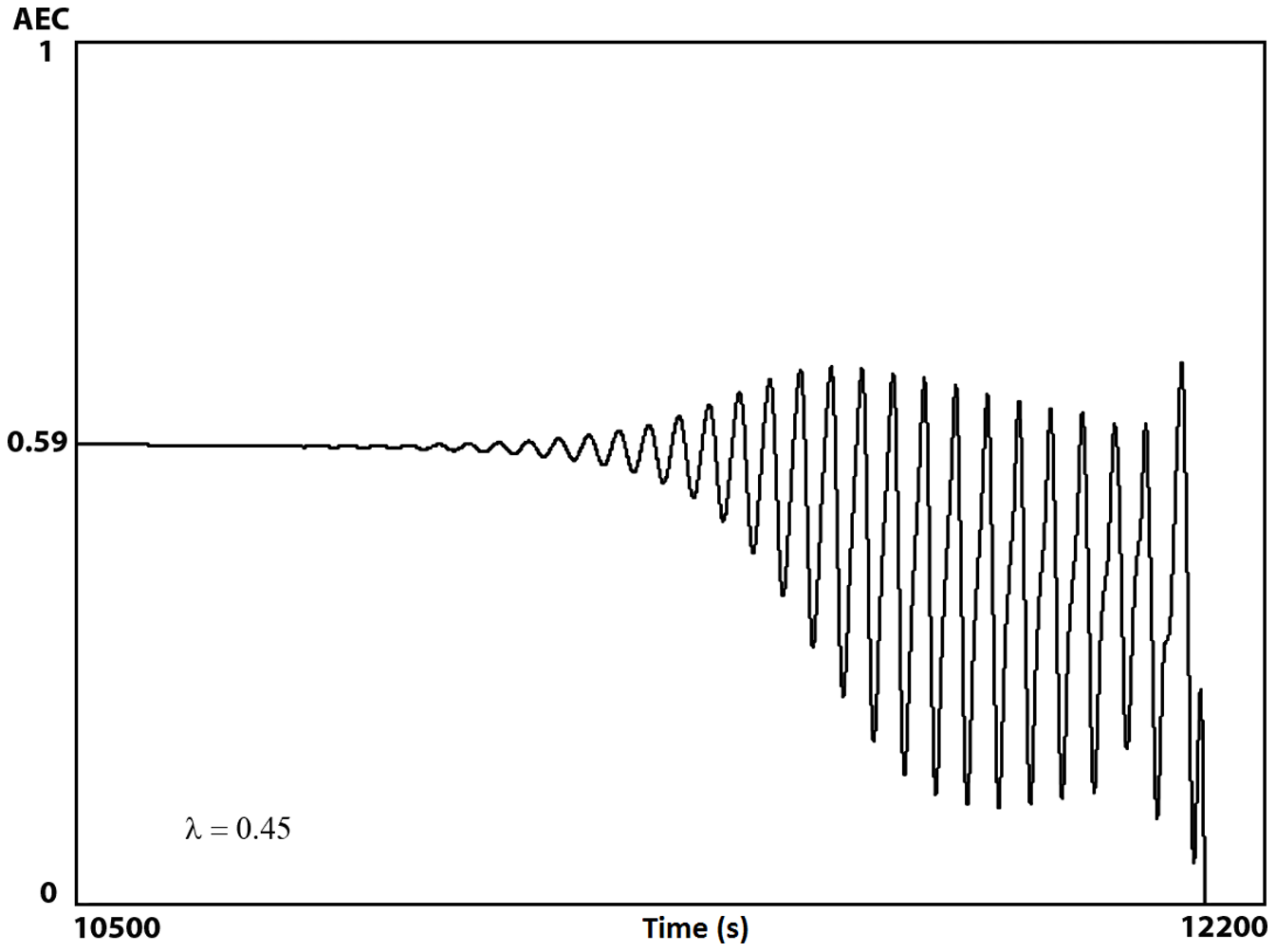
AEC dynamics under low production of ATP. AEC values as a function of time. At very small 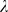 values (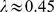), which represents a strong reduction of the ATP synthesis due to low substrate intake, the dynamic of the adenylate energy system shows a steady state behavior that slowly starts to descend, in a monotone way, up to reach the lowest energy values (AEC ∼0.59) at which the steady state loses stability and oscillatory patterns emerge with a decreasing trend. Finally, when the maximum of the energy charge oscillations reaches a very small value (AEC ∼0.28) the adenylate system suddenly collapses after 12,000 seconds of temporal evolution.

At very small 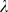 values, for 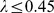, the AEC exhibits values below 0.6, which are gradually descending up to reach very small energy values, when the system finally collapsed (Figure 7) [97]–[101].

During decades, experimental studies have shown that when yeast cells are harvested, starved and then supplemented they exhibit significant metabolic oscillations.

**Figure 8:**
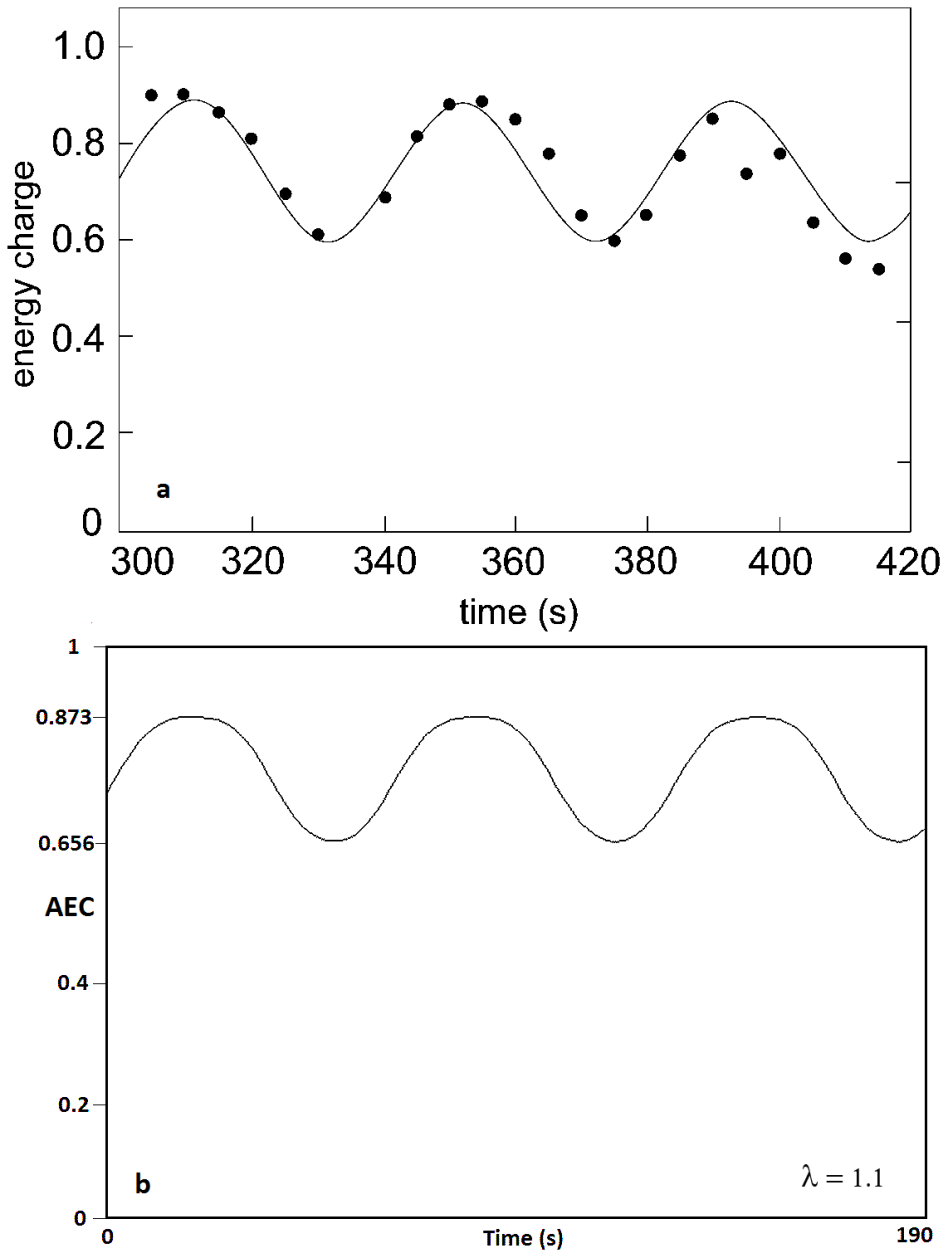
Experimental vs numerical results of AEC oscillations. Figure 7a illustrates a classical study of the intracellular adenine nucleotides in a population of intact cells belonging to the yeast *Saccharomyces cerevisiae* [30] which exhibits AEC rhythms, with max = 0.9, min = 0.6 and a period around 50 s. The authors fitted the experimental points to a sinusoidal curve. Figure 7b shows AEC oscillations belonging to our model at high values of ATP synthesis (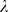 = 1.1), with max = 0.873, min = 0.656 and a period of 65 s.

Following these observations, we have compared our results with a classical study for oscillations of the intracellular adenine nucleotides in a population of intact cells belonging to the yeast *Saccharomyces cerevisiae* [30]. These cells were quenched 5 min after adding 3 mM-KCN and 20 mM-glucose at time intervals of 5 s. Figure 8a shows the dynamics of adenine nucleotide concentrations experimentally obtained, exhibiting AEC rhythms between 0.6 and 0.9 values (in the first and second oscillation) and a period of around 50 s. In addition, Richard and colleagues attempted to fit a sinusoidal curve through the experimental points [30]. Figure 8b shows an AEC oscillatory pattern at high values of ATP synthesis (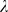 = 1.1), max = 0.873, min = 0.656 and a period of 65 s.

### Scenario II

In this second Scenario we have considered r_2_ as the control parameter, modeling time delays for ADP.

The numerical bifurcation analysis reveals that the temporal structure of the system (12) is complex, with several Hopf bifurcations emerging as well as secondary bifurcations of Neimark-Sacker type (torus), along two branches of limit cycles (Figure 3 d–f).

Concretely, using the numerical continuation package DDE-Biftools [189], we find 5 Hopf bifurcations. Two pairs of Hopf bifurcations are connected in parameter space, that is, the branch of limit cycles born at one, ends at the other, and the fifth Hopf bifurcation gives a branch that extends up to the upper limit of the interval considered, that is, r_2_ = 120 s.

Gradually increasing r_2_ from 1 s, we find that the branch of stable steady states that exists at r_2_ = 1 s destabilizes at a first Hopf bifurcation occurring at r_2_∼20.12 s. This Hopf bifurcation is supercritical, which means that the emanating family of limit cycles is stable; this family remains stable until it disappears through the second (also supercritical) Hopf bifurcation at r_2_∼37.04 s, which allows the family of steady states to re-stabilize; it remains stable until a third supercritical Hopf bifurcation occurs at r_2_∼71.94 s, rapidly followed by another Hopf bifurcation, subcritical, at r_2_∼72.83 s.

This marks the beginning of the region where the system is multi-stable, with one stable steady state and (at least) one stable limit cycle. The last Hopf bifurcation, terminating the branch of limit cycle born at r_2_∼72.83 s, is subcritical.

The reason for the complex integral solutions in the Scenario II is the presence of several torus bifurcations detected along both branches of limit cycles in the region of r_2_ between 71 s and 110 s. We recall that a Torus bifurcation occurs on a branch of limit cycles when a pair of complex-conjugated Floquet multipliers, leave the unit circle (in the complex plane). This corresponds to the fact that this branch of limit cycles becomes unstable and the stable solution starts winding on an invariant torus, periodic or quasi-periodic. We detect four Torus bifurcations, corresponding to the appearance and disappearance of multi-frequency oscillations, at the following values of r_2_: r_2_∼73.22 s, r_2_∼74.66 s, r_2_∼85.66 s, and r_2_∼106.06 s. Note the following additional details about the Figure 3 d–f we made: branches of stable (resp. unstable) steady states are represented by solid (resp. dashed) black lines; branches of stable (resp. unstable) limit cycles are represented by the max of the oscillation in blue and the minimum in red and by solid (resp. dashed). Hopf bifurcation points are represented with black dots labeled H; Torus bifurcation points with blue dots labeled TR. The horizontal axis corresponds to the bifurcation parameters: 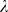 (Scenario I) and r_2_ (Scenario II). The vertical axis corresponds to the dependent variable's maxima along various computed branches.

**Figure 9:**
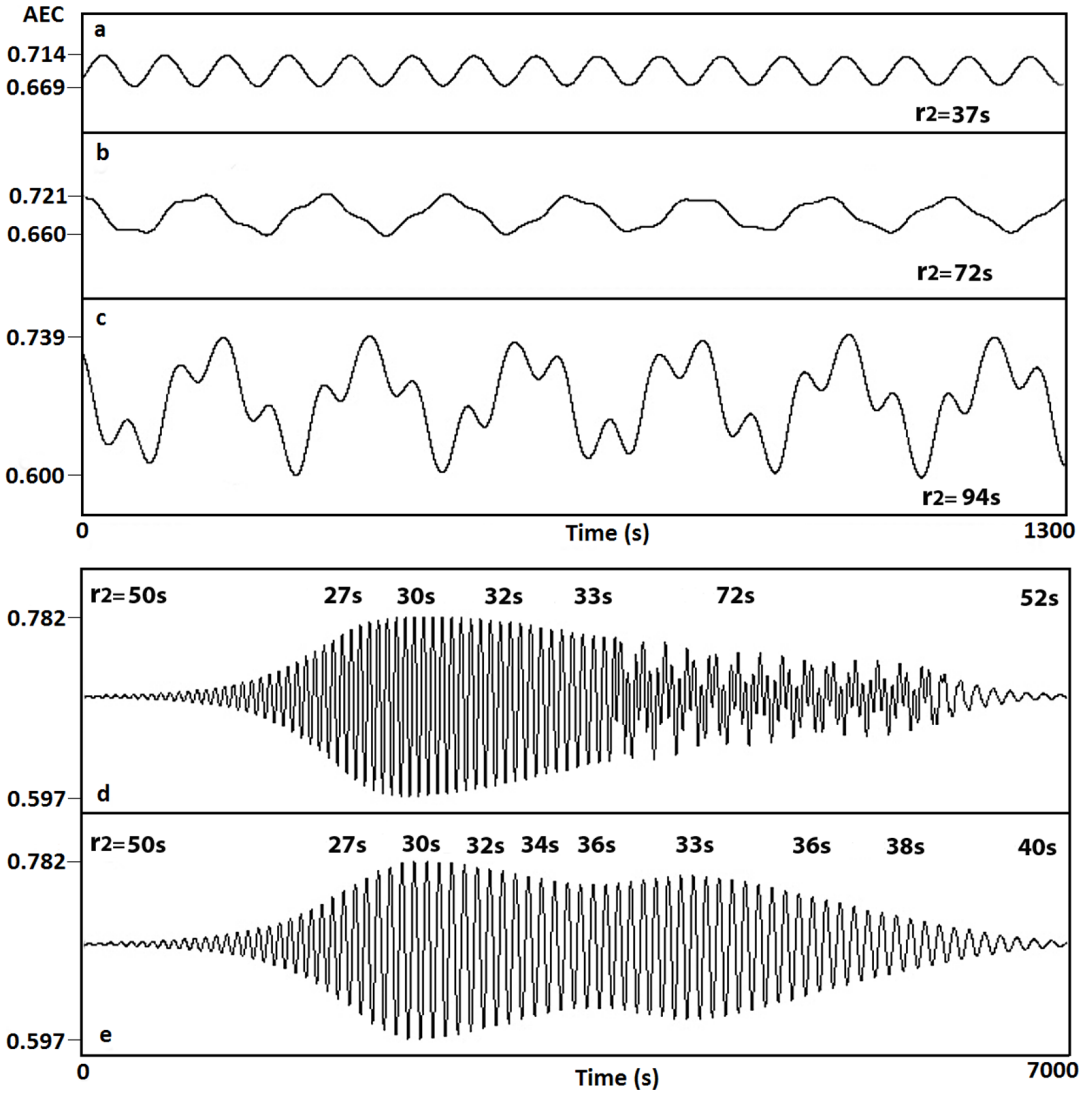
Emergence of oscillations in the AEC (Scenario II). Different oscillatory behavior appears when varying r_2_, controlling the ADP time delays. (a) For r_2_ = 37 s the AEC periodically oscillates with a very low relative amplitude of 0.045. (b–c) Existence of complex AEC oscillatory patterns for: (b) r_2_ = 72 s and (c) r2 = 94 s. (d–e) AEC transitions between different oscillatory behavior and steady state patterns for several r_2_ values. (d) 50 s, 27 s, 30 s, 32 s, 33 s, 72 s, 52 s. (e) 50 s, 27 s, 30 s, 32 s, 34 s, 36 s, 33 s, 36 s, 38 s, 40 s.

Figure 9 illustrates several examples of oscillatory patterns for the adenylate energy charge under different delay times. For r_2_ = 37 s the AEC periodically oscillates (Figure 9a). Increasing r_2_ up to 72 s (Fig. 9b) and up to 94 s (Fig. 9c) there exist complex AEC oscillatory patterns. Finally, AEC transitions between different oscillatory behavior and steady state patterns are observed for several r_2_ values (Figure 9d–e): (d) 50 s, 27 s, 30 s, 32 s, 33 s, 72 s, 52 s, (e) 50 s, 27 s, 30 s, 32 s, 34 s, 36 s, 33 s, 36 s, 38 s, 40 s. These r_2_ values and the respective integration times have been arbitrarily taken.

## Discussion

Energy is the fundamental element to maintain the turnover of the bio-molecular structures and the functional metabolic viability of all unicellular organisms.

The concentration levels of ATP, ADP and AMP reflect roughly the energetic status of cells, and a determined ratio between them was proposed by Atkinson as the adenylate energy charge (AEC) [96]. Under growth conditions, organisms seem to maintain their AEC within narrow physiological values, despite of extremely large fluctuations in the adenine nucleotide concentrations [96]–[101]. Intensive experimental studies have shown that the AEC ratio is preserved in a wide variety of organisms, both eukaryotes and prokaryotes (for details see Introduction section).

In order to understand some elements that determine the cellular energy status of cells we have analyzed a biochemical model conformed by some key essential parts of the adenylate energy system using a system of delay differential equations (12) in which the enzymatic rate equations of the main processes and all the corresponding physiological kinetic parameters have been explicitly considered and tested experimentally *in vitro* by other groups. We have used delay-differential equations to model the asynchronous metabolite supplies to the enzymes (substrates and regulatory molecules).

From the model results, the main conclusions are the following:

I. The adenylate energy system exhibits complex dynamics, with steady states and oscillations including multi-stability and multi-frequency oscillations. The integral solutions are stable, and therefore the adenine nucleotide concentrations (dependent variables of the system) can perform transitions between different kinds of oscillatory behavior and steady state patterns in a stabilized way, which is similar to that in the prevailing conditions inside the cell [15], [16].

II. The model is in agreement with previous experimental observations [15], [16], [30], showing oscillatory solutions for adenine nucleotides under different ATP synthesis conditions, at standard enzymatic concentrations, and for different ADP delay times.

III. In all the numerical results, the order of concentration ratios between the adenine nucleotides is maintained in a way that the highest concentration values correspond to ATP, followed by ADP and AMP which displays the lowest values, in agreement with the experimental data obtained by other authors [30], [109].

IV. During the oscillatory patterns, ATP and ADP exhibit anti-phase oscillations (the maxima of ATP correspond with the minima of ADP) also experimentally observed in [30].

V. As a consequence of the rhythmic metabolic behavior, the total adenine nucleotide pool exhibits oscillatory patterns (see experimental examples of this phenomenon in [188], [190], as well as the Gibbs free energy change for ATP hydrolysis (see [30]). In agreement with these results, we have found that the oscillation for the Gibbs free energy has a maximum and minimum values per period of −37.64 kJmol^−1^ and −33.99 kJmol^−1^, the same order of magnitude as in experimental observations (about −50 kJmol^−1^ in rat hepatocytes) [191].

VI. The adenylate energy charge shows transitions between oscillatory behaviors and steady state patterns in a stabilized way. We have compared an integral solution of our model with a classical study of intracellular concentrations for adenine nucleotides in a population of intact cells belonging to the yeast *Saccharomyces cerevisiae* and the model fits well with these data [30].

VII. The adenylate energy charge (AEC) does not substantially change during the simulations, indicating that is strongly buffered against the perturbations, in agreement with experimental data [97]–[101].

We want to remark that we have observed oscillatory patterns in the AEC, in the sum of ATP plus ADP and in the total adenine nucleotide pool but with very low amplitude, what might make difficult the experimental observation with traditional methods.

In fact, it is not clear yet what methodologies are the most appropriate to monitor the values of adenine nucleotides [192]. Although bioluminescence assays and high-performance liquid chromatography are the ones most commonly used for most of the studies [151], [192], these procedures are discontinuous and do not allow to observe real-time variations at short temporal periods. Moreover, adenosine nucleoside levels are critically dependent on sample manipulation and extraction by traditional methods. It has been demonstrated that even short lapses in sample preparation (2 min) can dramatically affect results [193].

It has been assumed for a long time that the temporal evolution of ATP, ADP and AMP concentrations present permanent steady state solutions and that, consequently, cells maintain the AEC as a constant magnitude (*homeostasis*). But this conservation is hard to be fulfilled for open systems.

Recently, the use of nanobiosensors has shown to be able to perform real-time-resolved measurements of intracellular ATP in intact cells; the ATP concentration is indeed oscillating, either showing a rhythmic behavior or more complex dynamics with variations over time, but importantly, the ATP concentration is never constant [15], [16].

As a consequence of our analysis we suggest that the appropriate notion to describe the temporal behavior of ATP, ADP and AMP concentrations is *homeorhesis* i.e. the non-linear dynamics of the adenylate energy system shape in the phase space permanent transitions between different kinds of attractors including steady states (in cellular conditions correspond to quasi-steady states) and oscillating attractors, which represent the sets of the asymptotic solutions followed by the adenine nucleotide variables.

*Homeorhesis* is substantially different to *homeostasis*, which basically implies the ability of the system to maintain the adenine nucleotide concentrations in a constant state.

The concept of *homeostasis* was first suggested by the physiologist Walter Cannon [194] in 1932, but its roots are found back to the French physiologist Claude Bernard who argued that an alleged constancy of the internal medium for any organism results from regulatory processes in biological systems [195], [196]. For a long time, the notable idea by Claude Bernard of constancy in the internal medium has paved the route of how cellular processes behaved. However, this constancy seems to be apparent.

In mid-twentieth century, the term *homeorhesis* was suggested to be a substitute of *homeostasis* by the prominent biologist Conrad Waddington [197], [198] to describe those systems which return back to a specific dynamics after being perturbed by the external environment, thus opposite to *homeostasis*, in which the system returns back to a fixed state. Later, that concept of *homeorhesis* was mathematically applied in distinct biological studies [199]–[204].

Rather than a permanent physiological stable state (*homeostasis*), living systems seem to be characterized by changing energy dynamics (*homeorhesis*).

In our numerical study, the temporal dynamics for the concentrations of ATP, ADP and AMP are determined by the adenylate energy system, and these adenosine nucleotide dynamics present complex transitions across time evolution suggesting the existence of homeorhesis.

In addition, we have observed that the values of the AEC do not substantially change during the simulations indicating that is strongly buffered against the perturbations. Recall that the AEC represents a particular functional relationship between the concentrations of adenine nucleotides.

As indicated in the introduction section, intensive experimental measurements under growth cellular conditions have shown that AEC values between 0.7 and 0.95 are invariantly maintained in practically all classes of cells which seems to represent a common key feature to all cellular organisms.

Hence, there appear to be two essential elements in determining the cellular energy level: first, the adenylate energy system originates complex transitions over time in the adenosine nucleotide concentrations so that there is no homeostasis for energy; second, it emerges a permanent relationship among the dynamics of adenine nucleotide concentrations (AEC values between 0.7 and 0.95), which seems to be strictly fulfilled during all the metabolic transformations that occur during the cell cycle.

These facts make possible to suppose that the cell is an open system where a given magnitude for energy is not conserved but there exists a functional restriction on the possible values that can adopt the adenine nucleotide concentrations.

At least, there seems to be a determinate function relating the adenine nucleotide values which appears to be invariant to all metabolic transformations occurring along the cell cycle. This invariant function, which it would define the real cellular energy state, might possibly have a complex attractor in the phase space since complex dynamic transitions in the adenine nucleotide concentrations have been observed *in vivo* [16], but these hypotheses need deserve further investigation.

Our interpretation to explain the essential elements of the cellular energy charge is that, in addition to the dynamical system which originates the complex transitions in the adenosine nucleotides, there exists an invariant of the energy function which restricts the values that adenylate pool dynamics can take, and the equation of Atkinson is the manifestation of that invariant function.

The main biological significance of the invariant energy function would be that under growth cellular conditions, the adenylate pool must be highly phosphorylated keeping the rate of adenylate energy production similar to the rate of adenylate energy expenditure.

Cell is a complex non-linearly open system where there is not a specific energy value which is conserved, but rather dynamic forms of change for energy. Unicellular organisms need energy to accomplish the fundamental tasks of the cell metabolism: today, in the post-genomic era, the understanding of the elemental principles and quantitative laws that govern the adenylate energy system is crucial to elucidate some of the fundamental dynamics of cellular life.
